# Relationships between oral health-related self-efficacy, oral health literacy, social support, and illness perception among patients with periodontitis: a moderated mediation model

**DOI:** 10.2340/aos.v85.45416

**Published:** 2026-02-06

**Authors:** Jinfeng Li, Rui Liu, Huan Zhang, Min Cao, Lei Che, Yixin Wang, Yunchao Fan

**Affiliations:** a State Key Laboratory of Oral & Maxillofacial Reconstruction and Regeneration, National Clinical Research Center for Oral Diseases, Shaanxi Key Laboratory of Stomatology, Department of Nursing, School of Stomatology, The Fourth Military Medical University, Xi’an, Shaanxi, China; b Department of Nursing, School of Nursing, The Fourth Military Medical University, Xi’an, Shaanxi, China; c State Key Laboratory of Oral & Maxillofacial Reconstruction and Regeneration, National Clinical Research Center for Oral Diseases, Shaanxi Key Laboratory of Stomatology, Department of Periodontology, School of Stomatology, The Fourth Military Medical University, Xi’an, Shaanxi, China; d State Key Laboratory of Oral & Maxillofacial Reconstruction and Regeneration, National Clinical Research Center for Oral Diseases, Shaanxi Key Laboratory of Stomatology, Department of Prosthodontics, School of Stomatology, The Fourth Military Medical University, Xi’an, Shaanxi, China

**Keywords:** Periodontitis, oral-health literacy, oral health-related self-efficacy, mediation analysis, moderated mediation analysis

## Abstract

**Objective:**

To assess oral-health literacy and test a conceptual model of the relationships among oral health-related self-efficacy, social support, illness perception, and oral-health literacy in patients with periodontitis.

**Materials and methods:**

This cross-sectional study recruited 230 eligible adult periodontitis patients by convenience sampling at the Department of Periodontology, Fourth Military Medical University (Xi’an) from March to July 2024. Participants completed validated Chinese versions of the short-form Health Literacy Dental Scale, the Self-efficacy Scale for Self-care, the Social Support Rating Scale, and the Brief Illness Perception Questionnaire. Data analysis included descriptive statistics, nonparametric tests, correlation analysis, and Hayes’s PROCESS macro (Model 4 and Model 7) to test for social support’s mediation and illness perception’s moderation.

**Results:**

The study included 230 participants (121 males, 52.6%; 109 females, 47.4%), with a mean age of 39.27 ± 11.85 years. The health literacy score for periodontitis patients was 42.00 (34.00, 52.00). The direct effect of oral health-related self-efficacy on oral-health literacy was significant (β = 0.2367, 95% CI [confidence interval] = [0.1176~0.3558]), and social support played a partial mediating role between oral health-related self-efficacy and oral-health literacy (β = 0.0526, 95% CI = [0.0153~0.0980]). Illness perception did not significantly moderate the relationship between oral health-related self-efficacy and social support (β = -0.0061, 95% CI = [-0.0133, 0.0010]).

**Conclusions:**

This study assessed oral-health literacy at a moderate level in periodontitis patients. The tested model was partially supported: self-efficacy directly improved oral-health literacy, and social support partially mediated this relationship. Illness perception did not moderate the pathway. Therefore, self-efficacy and social support are primary targets for improving oral-health literacy.

## Introduction

Periodontitis is a prevalent chronic inflammatory disease of the oral cavity and is caused by the accumulation of dental plaque, which leads to gingival recession, alveolar bone loss, and eventual tooth loss [1]. An increasing number of studies have established links between periodontitis and respiratory diseases, cardiovascular conditions, and diabetes mellitus [2]. Findings from China’s Fourth National Report on Dental Epidemiology revealed a decline in adult periodontal health between 2005 and 2015, underscoring periodontal disease as a public health issue [3]. Given these trends, contemporary periodontal care strategies increasingly emphasize not only clinical management but also behavioral and psychosocial interventions [4]. Oral-health literacy, as a key behavioral determinant of oral health, plays a pivotal role in promoting health behaviors, preventing disease, and improving health outcomes [5]. Therefore, assessing oral-health literacy in patients with periodontitis is critical for developing targeted prevention and management strategies.

Oral-health literacy refers to an individual’s ability to seek, understand, and utilize oral-health information to make appropriate oral health-related decisions [6]. Patients with adequate oral-health literacy can better recognize symptoms, comprehend disease-related issues, and adhere to recommended oral health care practices, thereby achieving better oral-health outcomes [7].

Self-efficacy refers to the confidence of individuals in their ability to take actions to maintain health and alleviate symptoms [8, 9]. Paasche-Orlow developed a framework that links health literacy to health outcomes, hypothesizing that self-efficacy connects health literacy and self-management behaviors [10]. This proposed link is supported by evidence. For example, a study from the United States demonstrated that patients with stronger numeracy skills displayed higher self-efficacy, which in turn improved adherence to diabetes medications and enhanced glycated hemoglobin control [11]. However, not all studies have reached consistent conclusions. Another investigation into diabetic populations did not identify a significant relationship between health literacy and self-efficacy [12]. This discrepancy suggests that the relationship may be influenced by population characteristics or study design, highlighting the need for further validation in patients with periodontitis.

Social support encompasses the social resources and assistance individuals receive from their social networks, including family, friends, colleagues, and community organizations [13]. Studies have shown that social support provides emotional comfort, instrumental aid, and valuable information, thereby increasing self-efficacy in health behaviors and facilitating health behavior change [14, 15]. Furthermore, individuals with lower health literacy tend to participate less in social activities and have more limited access to health-related information, which may hinder the improvement of their health literacy [14].Therefore, social support may serve as a mediator in the relationship between self-efficacy and health literacy. In other words, self-efficacy may influence health literacy by increasing an individual’s ability to obtain and utilize social support.

Illness perception refers to the emotional and cognitive representations individuals form about their disease [16]. According to the Common-Sense Model of Illness Perception [17], more positive illness perceptions are associated with greater confidence in initiating and sustaining health-promoting behaviors [18]. Moreover, patients who receive higher levels of social support tend to hold more optimistic views about disease management and treatment outcomes, reporting less symptomatic distress and demonstrating more active engagement in the therapeutic process [19]. On the basis of this theoretical foundation, we pose the following question: does illness perception moderate the relationship between oral self-efficacy and social support in periodontitis patients?.

Although some studies have explored the relationship between self-efficacy and health literacy [20], few have focused on a moderated mediation model involving oral health-related self-efficacy, social support, illness perception, and oral-health literacy among patients with periodontitis. To address this gap, this study formulated the following hypotheses:

Hypothesis 1 (H1): Oral health-related self-efficacy is correlated with oral-health literacy. Hypothesis 2 (H2): Social support mediates the relationship between oral health-related self-efficacy and oral-health literacy.

Hypothesis 3 (H3): Illness perception moderates the first stage of the mediating effect, specifically by exerting a moderating effect on the association between oral health-related self-efficacy and social support.

## Materials and methods

### Study design and sample

A cross-sectional survey was conducted using convenience sampling among periodontitis patients at the dental hospital of the Department of Periodontology, School of Stomatology, the Fourth Military Medical University in Xi’an, China, from March 2024 to July 2024. The study protocol was approved by the Ethics Committee of the School of Stomatology, the Fourth Military Medical University (No. KQ-YJ-2025-236). All procedures were conducted in accordance with the ethical principles of the Declaration of Helsinki.

The inclusion criteria were as follows: (1) Diagnosed with periodontitis [21]; (2) aged 18 years or older; (3) absence of psychiatric disorders; and (4) complete medical records and voluntary participation in this study. The exclusion criteria were as follows: (1) Patients who were unable to complete the study questionnaire; (2) those with concurrent oral diseases; and (3) pregnant or lactating women.

It has been suggested that the sample size should be at least five times the total number of variables [22]. A total of 29 variables were included in this study: clinical information (13 variables), oral-health literacy (7 variables), social support (3 variables), oral health-related self-efficacy (3 variables), and illness perception (3 variables). Considering a 20% invalid response rate, the minimum required sample size was set at 182 to ensure an adequate number of valid questionnaires (calculation: 29 × 5/(1–20%)).

### Efforts to address potential sources of bias

To ensure data validity and reduce potential bias, several measures were implemented. Selection bias was controlled by applying strict, predefined eligibility criteria to all potential participants. Diagnoses were confirmed with Florida Probe measurements and radiographic records. Non-response bias was minimized by clearly explaining the study’s purpose and procedures to eligible individuals and by obtaining written informed consent before questionnaire distribution. All paper questionnaires were checked for completeness at the time of collection. In addition, all data collectors received uniform training to ensure consistent procedures and accurate data recording.

### Assessment tools

#### Demographic and clinical information

Demographic and clinical information was collected using self- designed questionnaires. These questionnaires were developed on the basis of a literature review and the Fourth National Report on Dental Epidemiology [3] and were subsequently revised and supplemented under the guidance of clinical health professionals.

#### Oral-health literacy

The short-form Health Literacy Dental Scale (HeLD-14), developed by scholar Jones and translated into Chinese by Jones et al. [23] and Yan et al. [24], was utilized to evaluate the level of oral-health literacy among patients with oral diseases. This scale consists of 14 items across seven dimensions: communication, access, receptivity, understanding, utilization, economic barriers, and support. Each item is rated on a 5-point scale ranging from 0 to 4. Higher scores indicate greater ability to perform oral health-related tasks, whereas lower scores suggest limited capacity and a lower level of oral-health literacy. In this study, the Cronbach α was 0.794.

#### Oral health-related self-efficacy

The Self-efficacy Scale for Self-care (SESS), originally developed by Kakudate et al. [25, 26] and translated into Chinese by Wu et al. [27], was utilized to assess the confidence of individuals in their oral health care behaviors. The SESS includes 15 items in three dimensions: self-efficacy for consulting a dentist, self-efficacy for brushing one’s teeth, and self-efficacy for maintaining one’s dietary habits. Scores ranged from 15 to 75, with scores of 15–53 categorized as low self-efficacy, 54–59 as medium self-efficacy, and 60–75 as high self-efficacy. In this study, the Cronbach α coefficient was 0.820.

#### Social support

Social support was assessed by the Perceived Social Support Rating Scale (SSRS) developed by Xiao [28], which is a widely used tool for evaluating social support in China. The SSRS includes 10 items categorized into three dimensions: objective support (consisting of 3 items), subjective support (encompassing 4 items), and utilization of support (with 3 items). With a higher score indicating a greater level of social support, the Cronbach α coefficient for this study was 0.781.

#### Illness perception

Illness perception was measured using the Brief Illness Perception Questionnaire (BIPQ), developed by Broadbent and translated into Chinese by Broadbent et al. [29] and Mei et al. [30]. The BIPQ consists of nine items that cover cognitive perceptions, emotional aspects, and the comprehension of illness. Items 1 through 8 utilize a 0–10 Likert scale, while the ninth item is an open-ended question that prompts participants to list the three primary perceived causes of their illness. A higher score indicates a greater perceived psychological burden of illness. Cronbach’s α for the BIPQ in this study was 0.782.

### Data analysis

SPSS version 26.0 was used for all the data analyses. Prior to analysis, the dataset was cleaned to ensure the absence of outliers and missing data. Kolmogorov–Smirnov tests revealed that the oral-health literacy data significantly deviated from normality (*p* < 0.05). Data for oral-health literacy and oral health- related self-efficacy are presented as medians and interquartile ranges (IQRs), whereas data for social support and illness perception are presented as the means and standard deviations (SDs), which are consistent with their distributions. In univariate analyses, the Kruskal–Wallis H test or Mann–Whitney U test was employed to evaluate differences in oral-health literacy across various demographic and clinical groups, given the nonnormal distribution of the data (Table 1). Pearson or Spearman correlation analysis was used to evaluate the relationships between the variables and oral-health literacy, depending on data distribution (Table 2).

**Table 1:** Oral health literacy scores across demographic and clinical characteristics of periodontitis patients (N = 230).

Variable	*N* (%)	Oral health literacy		
		*M* (P25, P75)	Test value	*P* value
Age(years)				
< 35	91 (39.6)	47.00 (37.00, 53.00)	14.396^a^	0.001*
35~50	79 (34.3)	42.00 (35.00, 52.00)		
> 50	60 (26.1)	35.00 (30.25, 48.50)		
Gender				
Male	121 (52.6)	41.00 (33.50, 51.00)	1.104^b^	0.269
Female	109 (47.4)	44.00 (35.00, 53.00)		
Place of residence				
Urban	197 (85.7)	43.00 (35.00, 52.00)	1.291^b^	0.197
Rural	33 (14.3)	36.00 (31.00, 50.00)		
Education level				
High school or below	60 (26.1)	35.50 (31.00, 43.75)	17.813^a^	0.000*
Associate	45 (19.6)	45.00 (35.00, 51.50)		
Undergraduate or higher	125 (54.3)	47.00 (36.00, 53.00)		
Type of medical insurance				
Urban employee basic medical insurance	134 (58.3)	46.00 (35.00, 52.00)	6.218^a^	0.101
Urban resident basic medical insurance	31 (13.5)	42.00 (32.00, 54.00)		
New rural cooperative medical insurance	22 (9.6)	35.00 (29.50, 45.75)		
Government-funded medical care	43 (18.7)	41.00 (35.00, 52.00)		
Monthly household income, RMB (yuan)				
< 6000	75 (32.6)	40.00 (32.00, 51.00)	3.913^a^	0.141
6000~10,000	82 (35.7)	45.50 (34.75, 53.25)		
> 10,000	73 (31.7)	44.00 (35.50, 52.00)		
Categories of chronic illnesses				
0 types	185 (80.4)	42.00 (35.00, 52.00)	3.026^a^	0.220
1 type	41 (17.8)	46.00 (33.00, 54.00)		
More than 2 types	4 (1.7)	27.00 (18.50, 49.00)		
History of dental check-up				
Yes	170 (73.9)	46.00 (35.00, 53.00)	3.333^b^	0.001*
No	60 (26.1)	36.00 (31.00, 46.75)		
Periodic periodontal consultations				
Yes	71 (30.9)	48.00 (37.00, 55.00)	3.780^b^	0.000*
No	159 (69.1)	40.00 (32.00, 50.00)		
Professional dental guidance				
Yes	42 (18.3)	47.50 (35.75, 55.50)	2.005^b^	0.045*
No	188 (82.7)	42.00 (33.00, 51.00)		
Community dental guidance				
Yes	18 (7.8)	50.50 (39.75, 55.00)	2.286^b^	0.022*
No	212 (92.2)	42.00 (33.00, 51.75)		
Number of household members				
1~2	39 (17.0)	42.00 (33.00, 52.00)	1.322^a^	0.516
3~4	156 (67.8)	42.00 (32.25, 52.00)		
4>	35 (15.2)	43.00 (37.00, 52.00)		
Periodontitis stage				
Stage 1	71 (30.9)	41.00 (34.00, 51.00)	1.004^a^	0.800
Stage 2	116 (50.4)	44.00 (35.00, 52.00)		
Stage 3	25 (10.9)	41.00 (28.50, 52.00)		
Stage 4	18 (7.8)	42.50 (23.00, 53.00)		

Note: Oral health literacy scores were assessed using the short-form Health Literacy in Dentistry scale (HeLD-14). Data for continuous variables with non-normal distribution are presented as median (25th percentile, 75th percentile).

a Kruskal-Wallis H test;

b Mann-Whitney U test;

* *p* < 0.05.

**Table 2:** Descriptive statistics and bivariate correlations among oral health-related self-efficacy, oral health literacy, social support, and illness perception (N = 230).

Variable	X ± SD/M (P_25_, P_75_)	Oral health literacy	Oral health-related self-efficacy	Social support	Illness perception
Oral health literacy	42.00 (34.00, 52.00)	1			
Oral health-related Self-efficacy	59.00 (50.00, 66.00)	0.287**	1		
Social support	38.77 ± 7.91	0.251**	0.226**	1	
Illness perception	37.50 ± 12.88	-0.208**	-0.240**	-0.189**	1

Note: Data are presented as mean ± SD for normally distributed variables or as median (P25, P75) for non-normally distributed variables. Bivariate correlations are reported as Pearson’s *r* (for pairs of normally distributed variables) or Spearman’s ρ (otherwise).

** *p* < 0.01.

Variables that showed a significant association (*p* < 0.05) in the univariate analyses (demographic and clinical characteristics) were included as covariates in subsequent mediation effect analyses. The mediation effect and moderated mediation model were tested using the PROCESS macro developed by Hayes (2017). Model 4 (simple mediation) was used to examine the mediating role of social support in the relationship between oral health-related self-efficacy and oral-health literacy (Table 3). A significant mediation effect was indicated if the 95% CI (confidence interval) for the indirect effect excluded 0 (Table 4). Model 7 (moderated mediation) was tested to examine whether illness perception moderated the first stage of the mediation pathway (Table 5). This model specifically tested whether illness perception moderated the association between oral health-related self-efficacy and social support. A significant moderation effect was indicated if the 95% CI for the interaction term between self-efficacy and illness perception did not include 0. Bias-corrected 95% CI were calculated using 5000 bootstrap resamples to determine the significance of the mediation and moderated mediation effects.

**Table 3:** Testing social support as a mediator between self-efficacy and oral health literacy.

Outcome variable	Associate variable	*R*	*R* ^2^	*F*	β	*t*	*P*
Oral health literacy	Oral health-related self-efficacy	0.4873	0.2374	9.8759	0.2892	4.8184	0.0000***
Oral health literacy	Social support	0.5270	0.2777	10.6216	0.2926	3.5104	0.0005***
Oral health literacy	Oral health-related self-efficacy	0.5270	0.2777	10.6216	0.2367	3.9161	0.0001***
Social support	Oral health-related self-efficacy	0.3260	0.1063	3.7723	0.1796	3.8100	0.0002***

Note: Analyses controlled for age, education level, history of dental check-up, periodic periodontal consultations, professional dental guidance, and community dental guidance. **p < 0.01,

*** *p* < 0.001.

**Table 4:** Decomposition of effects in the mediation model of self-efficacy, social support, and oral health literacy.

	Effect	SE	*T*	*P*	LLCI	ULCI	RE (%)
Total effect	0.2892	0.0600	4.8184	0.0000***	0.1709	0.4075	
Direct effect	0.2367	0.0604	3.9161	0.0001***	0.1176	0.3558	0.82
Indirect effect	0.0526	0.0208	/	/	0.0153	0.0980	0.18

Note: Indirect effect: oral health-related self-efficacy → social support → oral health literacy. Effect, effect size; SE: standard error; CI: 95% confidence interval; RE: Relative mediation effect.

*** *p* < 0.001.

**Table 5:** Testing illness perception as a moderator of the path from self-efficacy to social support in the mediation model.

Associate variable	Outcome variable	β	*t*	*P*	LLCI	ULCI
Social support	Oral health literacy	0.2926	3.5104	0.0005***	0.1283	0.4568
Oral health-related self-efficacy	Oral health literacy	0.2367	3.9161	0.0001***	0.1176	0.3558
Oral health-related self-efficacy	Social support	0.3953	2.7254	0.0069**	0.1094	0.6811
Oral health-related self-efficacy * Illness perception	Social support	-0.0061	-1.6991	0.0907	-0.0133	0.0010

Note: Controlled for: age, education level, history of dental check-up, periodic periodontal consultations, professional dental guidance, community dental guidance. CI: 95% confidence interval.

** *p* < 0.01,

*** *p* < 0.001.

## Results

### Demographic and clinical characteristics

Of the 246 questionnaires distributed, 230 were completed and included in this analysis, yielding a response rate of 93.5%. The mean age of the participants was 39.27 ± 11.85 years, and the sex distribution was nearly balanced (52.6% male). Most participants were urban residents (85.7%) and had obtained an undergraduate-level education or higher (54.3%). With regard to health care profiles, urban employee basic medical insurance was the most common insurance type (58.3%), and monthly household income was relatively evenly distributed across the three predefined tiers. Comorbid chronic conditions were absent in 80.4% of the patients. While a high proportion of patients (73.9%) reported a history of routine dental check-ups, considerably fewer reported receiving regular periodontal consultations (30.9%) or professional oral health guidance (18.3%). With respect to disease severity, half of the cohort (50.4%) was diagnosed with stage II periodontitis. Their detailed characteristics are presented in Table 1.

### Bivariate correlations among all the variables

The scores serve as indicators of participants’ performance in the areas of oral-health literacy, oral health-related self-efficacy, social support, and the perception of illness. Correlation analysis revealed significant positive associations between oral-health literacy and both oral health-related self-efficacy (*r* = 0.287, *p* < 0.01) and social support (*r* = 0.251, *p* < 0.01). A positive correlation was noted between social support and oral health-related self-efficacy (*r* = 0.226, *p* < 0.01). A significant negative correlation was observed between the perception of illness and oral-health literacy (*r* = -0.208, *p* < 0.01). Among these correlations, the relationship between oral-health literacy and oral health- related self-efficacy showed the highest coefficient (*r* = 0.287, *p* < 0.01) (Table 2).

### Analysis of the mediation effect

**Figure 1:**
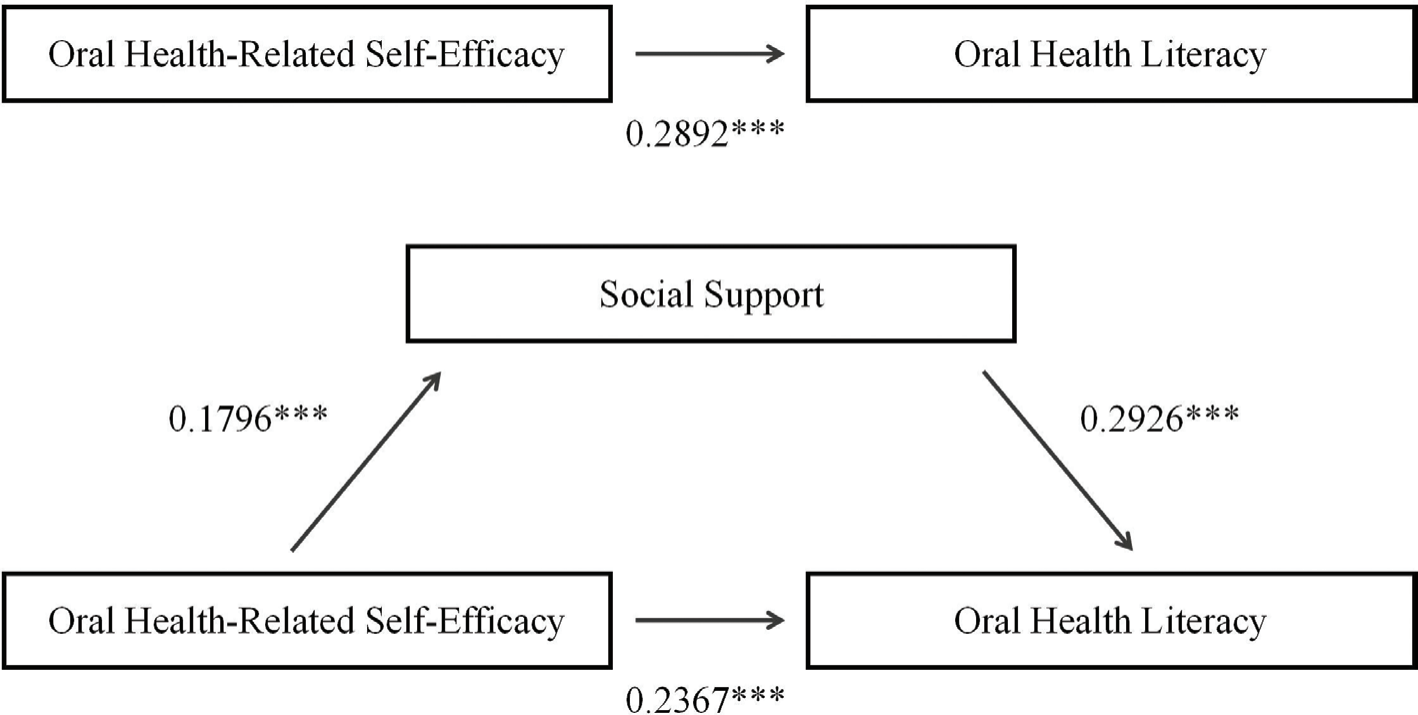
The proposed simple mediation model with social support mediating the relationship between self-efficacy and oral health literacy. Note. The relationship among effects is defined by the equation: Total Effect = Direct Effect + Indirect Effect, specifically: 0.2892 = 0.2367 + (0.1796 × 0.2926). All effects were statistically significant (total and direct effects: *p* < 0.001; indirect effect: 95% CI [0.0153, 0.0980]).

Model 4 from the SPSS macro developed by Hayes (2017) was used to examine the mediating role of social support in the relationship between oral health-related self-efficacy and oral-health literacy. After controlling for covariates such as age, education level, history of dental check-up, periodic periodontal consultations, professional dental advice, and community dental guidance, the total effect of oral health-related self-efficacy on oral-health literacy was found to be statistically significant (β = 0.2892, *t* = 4.8184, *p* = 0.0000). Upon the introduction of social support as a mediator, the direct predictive effect of oral health-related self-efficacy on oral-health literacy remained significant but decreased in magnitude (β = 0.2367, *t* = 3.9161, *p* = 0.0001). Significant correlations were observed between oral health-related self-efficacy and social support (β = 0.1796; *t* = 3.8100; *p* = 0.0002) and between social support and oral-health literacy (β = 0.2926; *t* = 3.5104; *p* = 0.0005). A bootstrap analysis was conducted. The results showed that the direct effect of oral self-efficacy on oral-health literacy remained significant (bootstrap 95% CI = 0.1176~0.3558). The indirect effect through social support was also significant (bootstrap 95% CI = 0.0153~0.0980) (Table 3, Figure 1), accounting for approximately 18% of the total effect (Table 4).

### Analysis of the moderating effects

**Figure 2:**
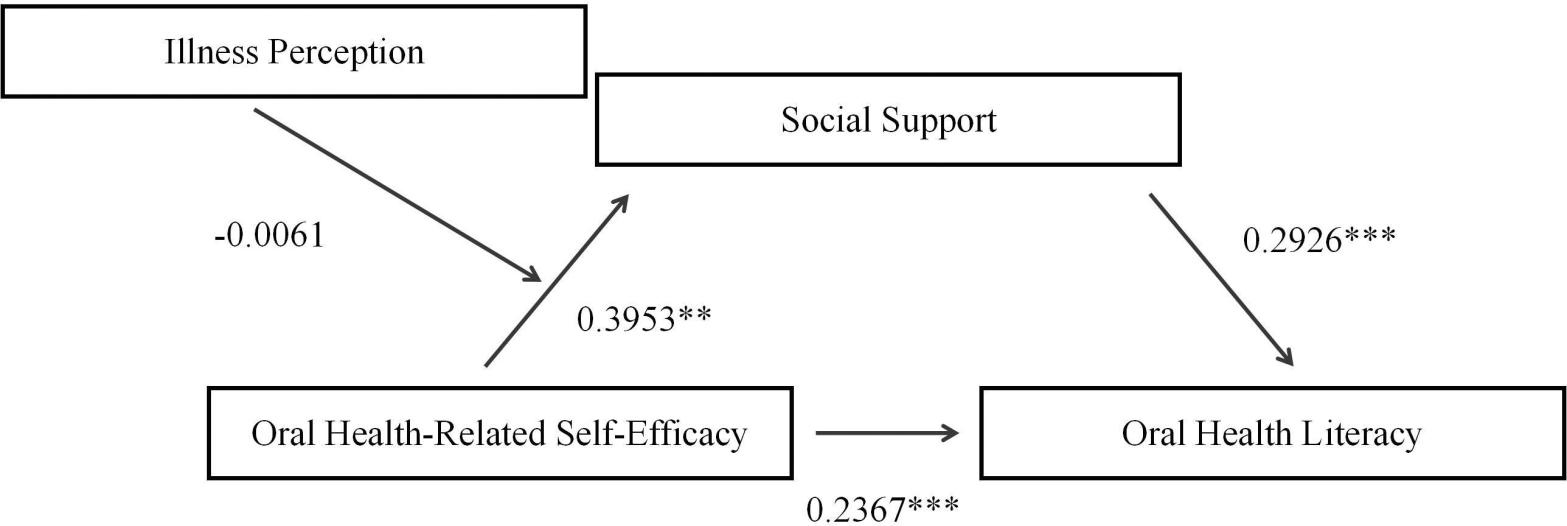
The moderated mediation model testing whether illness perception moderates the relationship between self-efficacy and social support. Note. The interaction term between self-efficacy and illness perception was not statistically significant (*p* = 0.0907), indicating that illness perception did not significantly moderate this relationship.

Model 7 of the SPSS macro developed by Hayes (2017) was employed to examine whether illness perception exerts a moderating effect on the relationship between oral health- related self-efficacy and social support. After controlling for covariates such as age, education level, history of dental check-up, periodic periodontal consultations, professional dental advice, and community dental guidance, both oral health-related self-efficacy (β = 0.2367, *t* = 0.9161, 95% CI = [0.1176, 0.3558], *p* = 0.0001) and social support (β = 0.2926, *t* = 3.5104, 95% CI = [0.1283, 0.4568] *p* = 0.0005) were found to significantly influence oral-health literacy. Furthermore, a significant relationship was observed between oral health-related self-efficacy and social support (β = 0.3953; *t* = 2.754; 95% CI = [0.1094, 0.6811]; *p* = 0.0069). However, the interaction term between oral health-related self-efficacy and illness perception on social support was not significant (β = -0.0061, *t* = -1.6991, 95% CI = [-0.0133, 0.0010], *p* = 0.0907) (Table 5, Figure 2).

## Discussion

Patients with periodontitis typically experience oral health problems. This underscores the critical need for them to acquire adequate oral-health literacy to enhance self-management and thereby improve health outcomes [5]. In the present study, health literacy score for periodontitis patients was 42.00 (34.00, 52.00), and periodontitis patients demonstrated a medium level of oral-health literacy, which is notably lower than the scores reported for orthodontic patients [31]. This difference highlights the potential challenges faced by periodontitis patients in managing their oral health. Consistent with previous research, educational level is significantly associated with health literacy [32]. Individuals with higher education exhibit higher levels of oral-health literacy, as higher educational attainment enables them to more easily understand professional health terminology and be better skilled at accessing valid information [33]. Similar to educational level, age was associated with a high level of oral-health literacy in this study, with younger patients generally demonstrating higher levels of oral-health literacy. This pattern may be attributed to younger individuals having broader access to information and greater initiative in acquiring new skills [34]. Furthermore, a history of regular dental check-ups, periodic periodontal consultations, professional dental guidance, and guidance from community sources were significantly associated with higher oral-health literacy. These channels allow for the accumulation of knowledge through ongoing health education by medical staff and can directly supplement patients’ health information reserves, all of which constitute important pathways for improving oral-health literacy [35]. Therefore, when designing oral-health literacy education programs, it is crucial to adopt accessible and straightforward methods tailored to the age and educational level of patients. A history of dental check-ups and periodic periodontal consultations significantly influences oral-health literacy. These findings underscore the importance of access to dental care and professional guidance in enhancing patients’ oral-health knowledge and skills. Consequently, it is important to encourage patients to attend periodic periodontal consultations as part of their oral-health treatments. Communities play a pivotal role in promoting oral-health literacy, and governments can facilitate this role by establishing counselling platforms and organizing regular oral health-related activities, such as lectures, to increase oral-health awareness and literacy among the population.

This study revealed significant correlations among oral health-related self-efficacy, social support, illness perception, and oral-health literacy. Social support partially mediated the relationship between oral health-related self-efficacy and oral-health literacy. However, illness perception did not significantly moderate the first half of the mediating effect between oral health-related self-efficacy and social support. Thus, Hypotheses 1 and 2 were supported, whereas Hypothesis 3 was not.

Previous studies have unequivocally demonstrated a close relationship between oral-health literacy, oral self-efficacy, and social support [31]. Both oral-health literacy and self-efficacy can motivate patients to manage oral diseases more actively and effectively and maintain oral health [36]. Oral-health literacy, such as mastering correct toothbrushing techniques, consulting dentists about specific measures to improve oral conditions, and evaluating the accuracy of obtained oral health information, is an essential skill for improving oral-health status [37].

Oral health-related self-efficacy is influenced by past dental experiences. Therefore, individuals with higher oral health-related self-efficacy are likely to have a history of successful experiences, which empowers them to improve their oral-health literacy more effectively [31]. Social support networks facilitate the dissemination of health information and the promotion of health behaviors among patients [38]. Patients with high health literacy potentially demonstrate superior communication skills, leading to increased interactions with others. Consequently, this advantage enables them to reshape their social networks and broaden their sources of social support; as previously discussed, societal support can augment the indirect experiences and exposure of individuals to verbal persuasion that encourages oral-health behaviors, ultimately improving their oral-health literacy [11]. The above discussion may explain the link between oral health-related self-efficacy and health literacy and the mediating role played by social support.

Therefore, when intervention programs aimed at enhancing oral-health literacy are designed, it is crucial to consider incorporating strategies to strengthen oral health-related self-efficacy and increase social support. Health care providers should encourage patients’ engagement in oral self-care, thereby increasing their confidence in managing periodontitis, supporting the development of social networks to maintain motivation for health behaviors, and encouraging active acquisition and understanding of oral health information, thereby enhancing self-efficacy and oral-health literacy for improved periodontal outcomes. Notably, the oral health-related self-efficacy score was relatively high (59.00 (50.00, 66.00)), which is consistent with previous reports [39].

Illness perception is a critical determinant of patient behavior [40]. Negative emotions often indicate a perceived threat from oral diseases, which can exacerbate disease progression. In this study, although illness perception did not significantly moderate the mediation model encompassing oral health-related self-efficacy and oral-health literacy, a significant negative correlation was observed between illness perception and oral-health literacy. That is, the more negative an individual’s perception of their illness is, the lower their level of oral-health literacy is likely to be. Therefore, assessing illness perceptions among individuals with oral diseases is crucial, as these perceptions can inform the development of strategies to guide oral health interventions and improve patient outcomes [41].

## Strengths and limitations

Although numerous studies have examined health literacy levels and their correlations, this study specifically focused on oral-health literacy levels in patients with periodontitis. We incorporated disease perception as an additional associated factor and elaborated on the mediator model that pertains to oral-health literacy. This study offers a novel methodological perspective that contributes to the exploration of factors influencing oral-health literacy among individuals with periodontitis. However, this study has several limitations. First, as a cross-sectional study, it cannot establish causal relationships between oral-health literacy and the other variables examined among patients. Second, all participants were recruited from high-ranking dental hospitals in China, which makes it uncertain whether the findings can be generalized to patients in other primary care settings. Additionally, the study population included only those patients who voluntarily sought medical attention, potentially excluding those with untreated dental disease. Furthermore, the assistance provided by researchers in completing the questionnaire may have inadvertently influenced the results.

## Conclusion

In this study, oral-health literacy was assessed at a moderate level in a cohort of periodontitis patients. Testing of the conceptual model revealed that oral health-related self-efficacy directly contributed to oral-health literacy, and that this relationship was partially mediated by social support. However, the hypothesized moderating effect of illness perception was not supported. These findings clarify a specific psychosocial mechanism and suggest that interventions aiming to improve oral-health literacy in this population should prioritize enhancing self-efficacy and strengthening social support networks.

## Data Availability

The anonymous data in this study are not available to share.
